# Liposomal Encapsulation for Systemic Delivery of Propranolol via Transdermal Iontophoresis Improves Bone Microarchitecture in Ovariectomized Rats

**DOI:** 10.3390/ijms18040822

**Published:** 2017-04-13

**Authors:** Benjamin Teong, Shyh Ming Kuo, Wei-Hsin Tsai, Mei-Ling Ho, Chung-Hwan Chen, Han Hsiang Huang

**Affiliations:** 1 Orthopaedic Research Center, Kaohsiung Medical University, Kaohsiung City 80708, Taiwan; benzblackcat@yahoo.com (B.T.); homelin@kmu.edu.tw (M.-L.H.); 2 Department of Biomedical Engineering, I-Shou University, Kaohsiung City 82445, Taiwan; smkuo@isu.edu.tw; 3 Department of Veterinary Medicine, National Chiayi University, Chiayi City 60054, Taiwan; s1050155@mail.ncyu.edu.tw; 4 Department of Orthopaedics, College of Medicine, Kaohsiung Medical University, Kaohsiung City 80708, Taiwan; 5 Department of Orthopedics, Kaohsiung Municipal Ta-Tung Hospital, Kaohsiung Medical University, Kaohsiung City 80145, Taiwan

**Keywords:** propranolol, liposome, bone loss, iontophoresis

## Abstract

The stimulatory effects of liposomal propranolol (PRP) on proliferation and differentiation of human osteoblastic cells suggested that the prepared liposomes-encapsulated PRP exerts anabolic effects on bone in vivo. Iontophoresis provides merits such as sustained release of drugs and circumvention of first pass metabolism. This study further investigated and evaluated the anti-osteoporotic effects of liposomal PRP in ovariectomized (OVX) rats via iontophoresis. Rats subjected to OVX were administered with pure or liposomal PRP via iontophoresis or subcutaneous injection twice a week for 12 weeks. Changes in the microarchitecture at the proximal tibia and the fourth lumbar spine were assessed between pure or liposomal PRP treated and non-treated groups using micro-computed tomography. Administration of liposomal PRP at low dose (0.05 mg/kg) via iontophoresis over 2-fold elevated ratio between bone volume and total tissue volume (BV/TV) in proximal tibia to 9.0% whereas treatment with liposomal PRP at low and high (0.5 mg/kg) doses via subcutaneous injection resulted in smaller increases in BV/TV. Significant improvement of BV/TV and bone mineral density (BMD) was also found in the fourth lumbar spine when low-dose liposomal PRP was iontophoretically administered. Iontophoretic low-dose liposomal PRP also elevated trabecular numbers in tibia and trabecular thickness in spine. Enhancement of bone microarchitecture volumes has highlighted that liposomal formulation with transdermal iontophoresis is promising for PRP treatment at the lower dose and with longer duration than its clinical therapeutic range and duration to exhibit optimal effects against bone loss in vivo.

## 1. Introduction

Bone is dynamically and constantly being remodeled to sustain a healthy skeleton. Trabecular bone is the main position of bone remodeling so it is also the main site of bone remodeling disease. This is also termed metabolic bone diseases [1]. In bone remodeling, osteoblasts are responsible for bone formation and osteoclasts undertake bone resorption. It was defined by Frost that bone remodeling is the balanced coupling of bone formation and resorption, resulting in a continuous replacement of old bone by new bone [2]. When people age, the coupling between bone resorption and formation shifts to an elevated rate of resorption and a declined rate of bone formation. Osteoporosis is a bone debilitating disease characterized by reduced bone mass and deterioration of bone microstructures which increases the risk of fracture [3]. In the aging female, bone loss increases after menopause due to deficiency of estrogen. Thus, the rise of elderly population leads to an increasing demand for effective treatment and prevention of osteoporosis.

Actions of anti-catabolic drugs focus on suppression of bone resorption to prevent and reverse age-associated reduction in bone mass and enhancement in bone fragility. Another challenging approach is to increase bone formation and osteoblastogenesis against age-related bone loss [4]. Despite findings of anabolic actions of some anti-osteoporetic therapy such as strontium ranelate [5], the only Food and Drug Administration (FDA) approved anabolic therapy for osteoporosis is parathyroid hormone [4]. However, it required daily subcutaneous injection which is inconvenient. Propranolol (PRP) is a nonselective β-adrenoreceptor blocker that is hydrophilic, highly lipid stable and has a low molecular weight. PRP was the first β-blocker approved and is known to be a widely used therapy for improving cardiovascular diseases such as ischaemic heart disease, and symptom control such as hypertension, arrhythmia and angina pectoris [6,7]. It has been reported that β-blockers such as PRP is able to enhance bone mass in murine models and epidemiological studies [8,9,10,11]. Reports showed that the greater preventive effect against ovariectomy (OVX) was observed in low-dose propranolol group in the OVX osteoporotic rats [9]. The work by Rodrigues et al. demonstrated that PRP decreased bone resorption in vivo and suppressed in vitro osteoclast differentiation and resorptive activity in the murine experimental periodontal model [12]. β2-adernergic agonists, on the other hand, exerted catabolic effects in primary human osteoblasts in vitro as these compounds increased bone resorption and decreased bone formation in vivo [13,14,15]. We have previously shown that PRP increases proliferation and osteoprotegerin (OPG) mRNA expression in primary human osteoblasts [13]. Nonetheless, PRP undergoes extensive hepatic first-pass metabolism and thus resulting in poor bioavailability [16]. These drawbacks may largely limit the potential anabolic effects of PRP on bone in vivo. Thus, we hypothesized that these disadvantages can be overcome, at least partially, by transdermal systemic delivery in combination with use of liposomal drug carriers through adjustment of administrative routes and controlled release of PRP.

Liposomes are defined as colloidal, vesicular structure composed by one or more lipid bilayers surrounding equal numbers of aqueous compartments. The unique structures have been proposed as a potential drug carrier for a wide variety of substances such as peptides and protein, hormones, enzymes, antibiotic, antifungal and anticancer agents [17,18,19,20,21,22]. Several liposome formulations are clinically used to combat cancer and infectious diseases and numerous are going through clinical trials [23]. Liposomes are biocompatible, biodegradable, non-toxic and non-immunogenic. In addition, liposomes increased stability of encapsulated drug and improved pharmacokinetic effects and therapeutic index of drugs [24,25]. Among classes of liposomes, phosphatidylcholine liposomes are the most widely used due to their relevance to the behavior of these components in cell membranes [26] as phospholipids are the core elements of liposomes [23]. The phospholipid 1,2-distearoyl-*sn*-glycero-3-phosphocholine (DSPC) has been used in preparation of liposomes for years and using DSPC, we have successfully fabricated liposomal PRP, which possessed similar stimulatory effects on human osteoblastic cell proliferation and differentiation [27]. Thus, DSPC liposomes were employed as a carrier for PRP encapsulation in this study.

The transdermal delivery route, such as iontophoresis, offers certain advantages including feasible controlled delivery or sustained release of drugs, avoidance of first pass-hepatic metabolism and a self-administered approach to drug delivery. Its non-invasive advantages over injective routes like intramuscular, intravenous and subcutaneous injections are safety and alleviation of pain. Therefore, transdermal delivery has been shown to be poised to provide alternatives and bring increasing impact on medicine [28]. Iontophoresis is characterized by uses of a small amount of physiologically acceptable direct current to drive ionized and unionized drugs across and into the skin. It has been indicated as a meritocratic and functionality-enhanced manner in the second-generation transdermal delivery systems [28,29]. Iontophoresis would increase the drug delivery efficiency to achieve therapeutic levels and might further allow modulation of delivery for individualized dosing. Other advantages of iontophoresis include enabling continuous or pulsatile drug delivery and provide better control over the amount of drug delivered [29]. To date, the potentials of using liposomes in collaboration with transdermal iontophoresis to enhance the permeability, bioavailability and effects of PRP against bone loss have not been investigated. The combination of liposome-encapsulation with iontophoresis may potentially synergize their merits to optimize the anabolic effects of PRP against bone loss. Thus, the hypothesis is that transdermal delivery of PRP combined with liposomal encapsulation is able to enhance the bioavailability and anabolic effects of PRP on bone. Comparison between transdermal delivery route and subcutaneous injective route in regard of improving bone microarchitecture in tibia and spine for evaluation of anabolic effects of pure and liposomal PRP in OVX osteoporetic rat model was revealed in the current study. The analysis and elevation of anabolic effects of liposomal PRP administered by iontophoresis on bone in vivo was verified in our work to highlight that application of liposomal encapsulation in combination with iontophoretic administration may therapeutically reinforce the actions of PRP against osteoporosis.

## 2. Results

### 2.1. Measurement of Animal Weight

**Figure 1:**
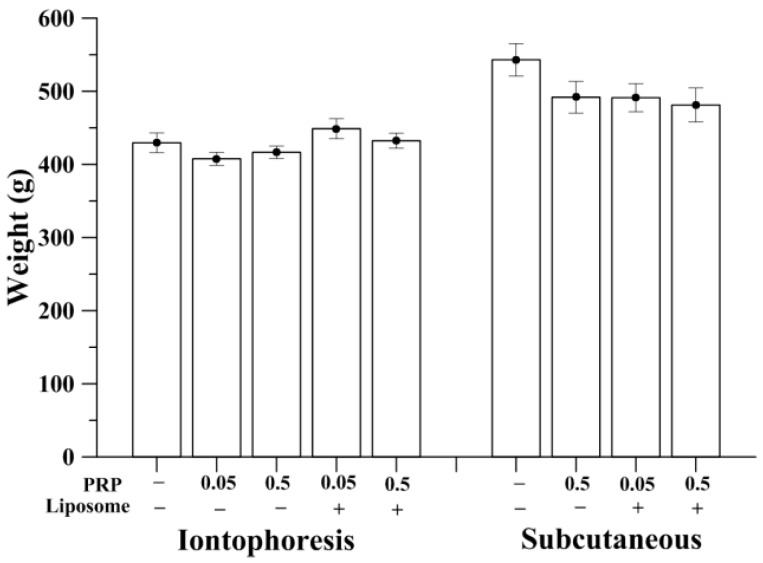
The average weight of rats in two administrative groups measured at the end of experiment (aged 9 months). Under identical drug form, the body weight of the subcutaneously injective rats (*n* = 8) was respectively greater than that given by iontophoresis (*n* = 13).

The weight of rats was measured at the end of the experiment. All animals gained weight but the mean body weight did not show statistically significant difference among groups administered with the same route, either by iontophoresis or subcutaneous injection. When identical drug form was given, the body weight of the rats with subcutaneous injection was respectively greater than that in the iontophoretic administration (Figure 1). Weight loss might be due to repeated restraint stress due to anesthesia during the iontophoretic process [30]. The processes of iontophorosis performed in rats were carefully monitored by veterinarians and experienced animal research technicians. During the administration period, no clinical signs of pain, salivation or abnormal behavior were found since Zoletil^®^ (Virbac, France) and xylazine have been appropriately administered. No significant changes in respiratory, physical responses to stimulation or neurological signs were observed in rats of iontophoresis groups. Control of subcutaneous injection groups (S-OVX) had the highest mean body weight, approximately 550 g whereas no obvious effects of pure or liposomal PRP on body weight were observed compared to S-OVX.

### 2.2. Trabecular Bone Analysis (Tibia)

**Figure 2:**
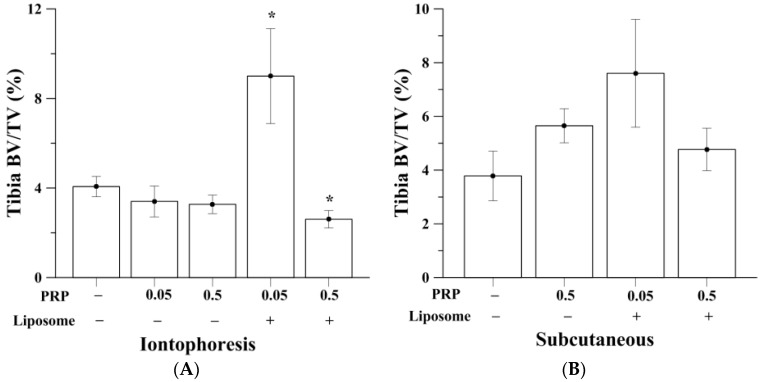

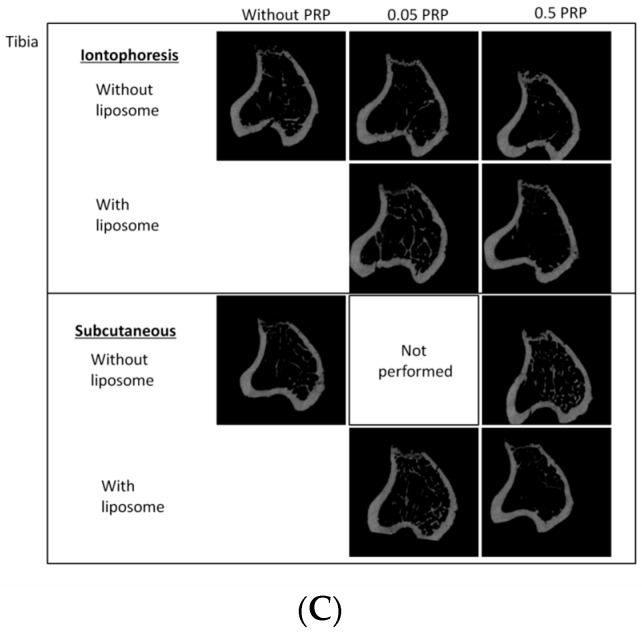
(**A**) Low-dose liposomal PRP (0.05 mg/kg) given by iontophoresis (*n* = 13) approximately doubled bone volume (BV)/total tissue volume (TV) percentage in tibia (* *p* < 0.05); (**B**) Subcutaneous injection of pure or liposomal PRP (*n* = 8) did not show significant effects on tibia BV/TV; (**C**) The micro-computed tomography (micro-CT) reconstructed images were in consistency with the BV/TV values of proximal tibia of the rat acquired in each experimental group.

**Figure 3:**
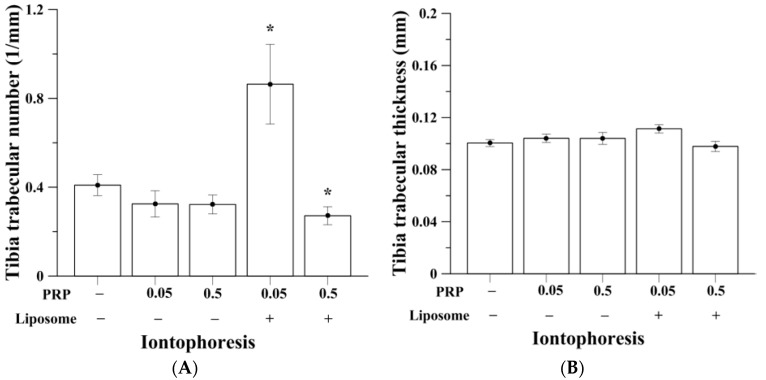
(**A**) Liposomal PRP at 0.05 mg/kg given via iontophoresis (*n* = 13) approximately doubled tibia trabecular number (* *p* < 0.05) and (**B**) slightly increased tibia trabecular number.

OVX induced bone loss in trabecular bone compartment of the tibia, was measured by micro-computed tomography (micro-CT) (Table 1). Figure 2A,B show the comparison of tibia bone volume (BV)/total tissue volume (TV) percentage among all experimental groups. Micro-CT reconstructed images of rats’ proximal tibia are shown in Figure 2C. OVX rats without PRP treatment (control of iontophoresis and subcutaneous groups; I-OVX and S-OVX) had BV/TV of approximately 4.1–4.5%, as opposed to a normal rat that would have BV/TV of approximately 40%, according to our previous study [31]. Treatment of pure PRP by iontophoresis, both at 0.05 and 0.5 mg/kg, were unable to rescue the bone loss leaving trabecular BV/TV of 3.41% ± 0.69% and 3.27% ± 0.41%, respectively. Animals that given iontophoretic liposomes-encapsulated 0.05 mg/kg PRP (I-0.05PRP/L) had the highest and over 2-fold enhancement in BV/TV (9.00% ± 2.04%) compared to I-OVX (*p* < 0.05). I-0.05PRP/L also significantly increased mean trabecular numbers in tibia (*p* < 0.05, Figure 3), indicating that liposomal encapsulation was necessary to either permeate drug across the skin barrier or to prolong in vivo PRP anabolic effects on bone. I-0.5PRP/L had BV/TV of 2.61% ± 0.39%, which was lower than I-0.05PRP/L, and even lower than that in I-OVX group. On the other hand, subcutaneous injection of pure 0.5 mg/kg and liposomal 0.05 or 0.5 mg/kg PRP resulted in insignificant increase of BV/TV percentage as compared with S-OVX group. Also, PRP of pure or liposomal dosage forms administered subcutaneously could not cause any significant increases in trabecular numbers and mean trabecular thickness in tibia (data not shown). I-0.05PRP/L group also showed slight and insignificant increase in bone mineral density (BMD) in tibia whereas subcutaneous injection of the two PRP forms did not cause significant changes in BMD in tibia in the OVX rats (Figure S1).

### 2.3. Trabecular Bone Analysis (Spine)

**Figure 4:**
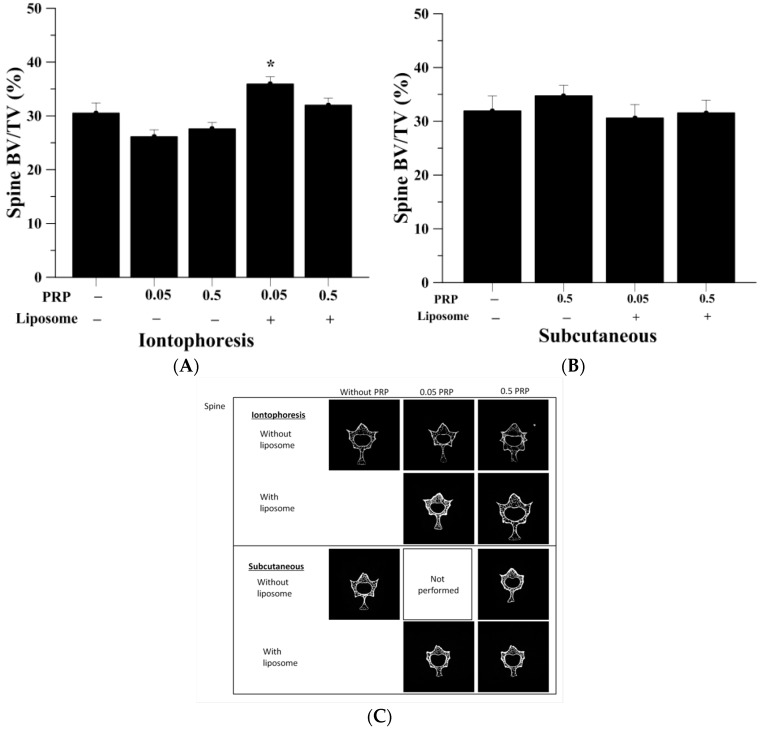
(**A**) Low-dose liposomal PRP (0.05 mg/kg) given by iontophoresis (*n* = 13) enhanced BV/TV in spine (* *p* < 0.05); (**B**) Subcutaneous injection PRP of pure or liposomal forms (*n* = 8) did not have significant effects on spinal BV/TV; (**C**) The micro-CT reconstructed images were in consistency with the BV/TV values of proximal tibia of the rat acquired in each experimental group.

**Figure 5:**
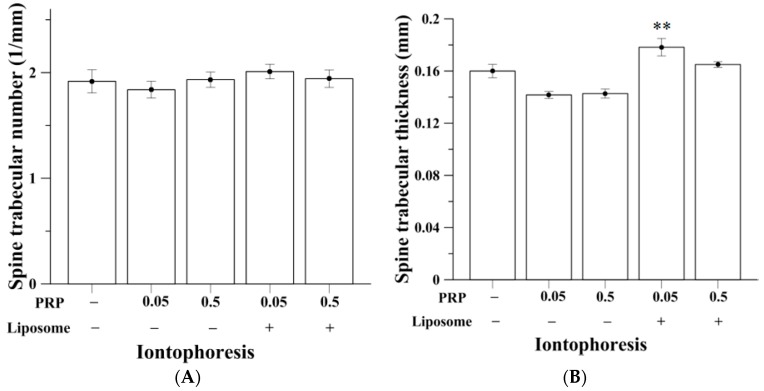
(**A**) Liposomal PRP (0.05 mg/kg) given by iontophoresis (*n* = 13) mildly increased spinal trabecular number; (**B**) Iontophoretic liposomes-encapsulated PRP (0.05 mg/kg) significantly increased spinal trabecular thickness (*n* = 13, ** *p* < 0.01).

The micro-CT analysis of trabecular bone in spine was specific at the fourth lumbar section (Table 2). A normal rat would have BV/TV of approximately 80%. As shown in Figure 4A,B, the OVX group had BV/TV of 30.51% ± 1.90% and 31.93% ± 2.79% after iontophoretic and subcutaneous administration respectively. Using iontophoretic manner, pure PRP at both doses were unable to increase spinal trabecular bone mass, retaining BV/TV of 26.13% ± 1.29% and 27.60% ± 1.18%, respectively. However, by employing liposomes as drug carrier, I-0.05PRP/L significantly increased the trabecular bone volume of spine, having BV/TV of 35.93% ± 1.35% (*p* < 0.05). There were no statistically significant differences between spinal BV/TV values of S-0.5PRP, S-0.05PRP/L and S-0.5PRP/L compared to S-OVX group (Figure 4B). Reconstruction images of rats’ fourth lumbar spine assessed by micro-CT are shown in Figure 4C. Meanwhile, significantly increased spinal trabecular thickness was also observed in the I-0.05PRP/L group (*p* < 0.01) (Figure 5). No significant differences in trabecular number and trabecular thickness were found between the experimental groups when pure or liposomal PRP were given subcutaneously (data not shown). Also, both I-0.05PRP/L and I-0.5PRP/L exhibited significant increases in BMD in spine whereas no significant differences were observed in BMD among the groups with subcutaneous administration (Figure S2).

### 2.4. Effects on Serum Calcium, Phosphorous and Cholesterol Level

Biochemical parameters in serum of all groups were shown in Table 3. I-0.1PRP/L group (*p* < 0.01) and I-0.5PRP/L groups showed small but significant decrease in serum calcium (*p* < 0.01) compared to I-OVX group. S-0.05PRP/L also had a slight but significant decrease in serum calcium (*p* < 0.05) compare with S-OVX. Both I-0.05PRP and I-0.05PRP/L presented with decreases in serum phosphorous concentration compared to I-OVX group (*p* < 0.001 and *p* < 0.01, respectively) as S-0.05PRP also showed a decrease in serum phosphorous compared to S-OVX (*p* < 0.001). No significant differences were found in serum sodium and potassium between experimental groups (data not shown). No significant differences were found in serum cholesterol level among groups.

### 2.5. Effects on Liver and Kidney Function

For serum glutamic oxaloacetic transaminase (SGOT) level, the group I-0.05PRP/L showed a significant reduction (*p* < 0.05) while I-0.5PRP/L showed a significant increase (*p* < 0.05) compared with that of I-OVX (Table 4). There were no significant differences in serum glutamic pyruvate transaminase (SGPT) level compared to OVX group via either delivery routes. Reduction of creatinine was observed in the four iontophoretic-PRP treated groups compared with I-OVX (*p* < 0.05 or *p* < 0.01). Higher blood urea nitrogen (BUN) level (*p* < 0.05) was observed in I-0.5PRP/L compared to I-OVX.

## 3. Discussion

Osteoporosis is characterized by decreased bone mass and deterioration of bone architecture, resulting in higher risk of fragility fractures which clinically present with the main consequence of osteoporosis [32]. To re-balance the shifted coupling of bone remodeling in the elderly and the postmenopausal woman, therapies for osteoporosis can be aimed at reducing the rate mineralized bone resorption and/or at enhancing the rate of bone formation. On the other hand, fabrication of lipid-based nanoparticles as drug carriers, such as liposomes, can display a number of merits, including minimum toxicity of the carrier, relatively low-cost and ability of increasing drug of interest to cross the skin barrier. In our previous work, pure PRP enhanced primary human osteoblast replication and OPG expression [13] while the stimulatory effects of PRP and liposomal PRP on the proliferation and differentiation in human osteoblastic cells [27]. Both studies support the proposal that the pure PRP and prepared liposomes-encapsulated PRP possess anabolic effects on bone formation in vivo so liposomal PRP potentially plays protective roles against bone loss in animal osteoporetic models [27]. Therefore, in this study we examined the effects of pure and liposomal PRP at high and low doses on bone administered via different delivery methods in osteoporetic OVX rats. Iontophoretic liposomes-encapsulated PRP at low dose was found to efficiently increase bone volume both in the proximal tibia and spine in the current study.

β-Blockers, including PRP, have main drawbacks such as first-pass metabolisms and higher frequency of administration. Transdermal delivery system like iontophoresis potentially circumvents these disadvantages [16]. Without liposomes as drug carrier, water soluble PRP alone is unable to effectively penetrate into the animal body via iontophoresis. The development of carriers for the delivery of PRP constitutes a promising approach to improving its therapeutic activities and reducing its side effects [23]. It is well known that liposomes are small, sphere-shaped, and enclosed compartments which separate an interior aqueous space from another by amphipathic phospholipid bilayer [23,33]. Such characteristics promote liposomes becoming an important role in transdermal drug delivery system. Liposomes themselves raise stability of encapsulated drug and increase pharmacokinetic effects and therapeutic index of drugs [25]. The combination of liposomal encapsulation with iontophoresis is thus able to potentially maximize therapeutic effects of PRP and rationally decrease its side effects. On the other hand, several epidemiological investigations have shown that β-blockers are related with decreased fracture risk or BMD [8,10,11,34], or decreased levels of the bone resorption marker, *C*-telopeptide [35]. We have previously shown pure PRP and liposomal PRP possess stimulatory effects on the proliferation and differentiation in human osteoblastic cells [27]. The current study is the first report to reveal that liposomes-encapsulated PRP at the dose 0.05 mg/kg (lower than therapeutic range 0.1–5 mg/kg [36,37,38]) and with less dosing frequency (twice a week, less than that of clinically therapeutic medication) in combination with administration of iontophoresis exerted anti-osteoporetic effects. This demonstrates that the crucial advantages on enhancing efficacy of drug in vivo achieved by liposomal encapsulation and transdermal iontophoresis. Yet, the controlled release behavior of PRP-loaded liposomes remains challenging in this study, which would require a more complex liposomal formulation.

We have successfully used micro-CT and these crucial parameters to find that the green tea phenol (-)-Epigallocatechin-3-gallate (EGCG) increases bone volume and trabecular thickness in tibia and spine in OVX rats [31]. In the current study, we further evaluated the anabolic effects of pure and liposomal PRP administered via iontophoresis and subcutaneous injection on osteoporetic bones using micro-CT analysis in OVX rats. Administration of PRP in animals is commonly delivered via subcutaneous injection. Our results indicated that such treatment route presented with insignificant changes in the trabecular bone mass in both tibia and spine. Meanwhile, a less invasive and transdermal drug delivery method, iontophoresis, was compared in this study in which PRP was delivered across the dermal layer. Direct injection of the drug offers immediate presence of drug effects whereas iontophoresis provide slow and continuous delivery of drug into body. Moreover, encapsulation of liposomes may further render PRP extended effects since the gradual release of PRP from liposomes within 48 h, which we found in vitro drug release assay [27] may be attained in vivo while our current data certainly validate so, at 0.05 mg/kg lower than commonly-used therapeutic doses and further prolong dosing duration to twice a week. Both pharmacological merits of drug delivery and modification describe above should be beneficial for improving this rapidly metabolized drug and for developing more effective treatment against metabolic and progressive diseases like osteoporosis in which pathophysiologically prolonged efficacy of treatment may result in better outcomes after the therapy was administered.

The release of water soluble PRP from DSPC liposome was basically by diffusion mechanism. Thus, higher drug encapsulation in liposome leads to higher release rate. Moreover, the flow of electric current used in iontophoresis might trigger or facilitate the release of PRP, as reported by D’Emanuele and Staniforth [39]. According to the present data, lower dosage of PRP (0.05 mg/kg) consistently have significant improvement on bone microarchitecture than higher dosage, which is similar with results shown by Rodrigues and Bonnet [9,12,38], although high dose 0.5 mg/kg PRP in the current study accounts for approximately medium-to-low dosing level in previous studies. In particular, in the current study the liposomal encapsulation and iontophoretic delivery showed beneficial properties for administration of low-dose PRP in vivo. In comparison with the treating recipes and the previous data via subcutaneous injection in the OVX rats [9,38], using less administrative frequency (twice a week) and lower dose of PRP than its general therapeutically cardiovascular recommendations brought to significant increases in bone formation in terms of BV/TV percentage, mean trabecular number, mean trabecular thickness and BMD in tibia and spine in the low-dose iontophoretic liposomal PRP group. These results suggested that liposomal encapsulation combined with iontophoresis is highly likely capable of increasing bioavailability and facilitating PRP pharmacologically anabolic efficacy on bone formation in vivo. Thus, the effective dose of PRP for enhancing bone formation at osteoporotic status can be further decreased and the dosing duration can be extended in vivo. Meanwhile, compared to subcutaneous or intravenous injection conducted in previous studies, iontophoresis used in our study is non-invasive, much safer and more convenient when administered in humans. Also, the present results somewhat resembled the previous data that higher doses (≥0.5 mg/kg) of pure or liposomal PRP administered via subcutaneous injection or iontophoresis that presented with lower anabolic effects against osteoporotic bone or even a decrease in BV/TV in OVX rats [9,38]. The reasons could be associated with that PRP plays as a role of partial agonist particularly at high concentration [7,40,41], resulting in catabolic effects of adrenergic β-agonist on bone in vitro and in vivo [13,14,15], although anti-bone resorptive effects of PRP were reported in a study using rat model of experimental periodontal disease [12]. The mechanism of partial agonist for PRP action, a β-blocker and inverse agonists for Gs-stimulated adenylyl cyclase, has been shown to also induce response for mitogen-activated protein kinases extracellular signal-regulated kinase (ERK) 1/2 therefore acting as dual efficacy ligands [40]. Moreover, it is noteworthy that since the low but effectively anti-osteoporotic dose (0.05 mg/kg) of iontophoretic liposomal PRP in the current study is lower than its therapeutic values (range 0.1–5 mg/kg) [36,37,38]. Therefore the generally used in adult and the pediatric for treating arrhythmias, hypertension, hemangioma and angina pectoris, the cardiovascular and other systemic effects of PRP on the tissues or organs beyond bone in vivo would be decreased as desired.

In addition, the current data revealed that liposomes-encapsulation cooperated with transdermal iontophoresis displayed most stable and systemic effects of PRP against bone loss in terms of BV/TV, mean trabecular thickness, mean trabecular number and BMD compared with subcutaneous injection with or without liposomal encapsulation. Subcutaneous injection resulted in inconsistent and insignificant effects on proximal tibia and spine in OVX rats. High-dose pure PRP given subcutaneously led to an insignificant increase in BV/TV in tibia and spine compared to the treatments administered with identical route (Figure 2B and Figure 4B). The subcutaneous groups with both dosage forms did not display any significant changes in BMD in tibia or spine (Figure S1 and 2). As to the other assessment parameters of bone, subcutaneous injection with low-dose liposomal PRP showed highest but insignificant enhancement in trabecular thickness and trabecular number in tibia (data not shown). These results indicated that liposomes-encapsulation united with transdermal iontophoresis is able to render low-dose liposomal PRP to stably and more systemically exert significant anabolic effects against bone loss in both tibia and spine in the OVX rat model. Our in vivo data are therapeutically valuable since with application of liposomal encapsulation and iontophoretic administration, the systemic adverse effects of PRP can be decreased and catabolic effects of PRP on bone can be avoided due to the decreased effective dose while systemic anabolic effects of PRP on bone formation were apparently retained and exhibited. Therefore, the current results of lower dosage, longer administrative duration and effective in enhancing bone formation implicated the liposomal form of PRP with iontophoretic route may be applied to clinical use.

The effects of administration of iontophoretic-PRP and subcutaneous-PRP at high (0.5 mg/kg) and low (0.05 mg/kg) doses on body weight, serum calcium, serum phosphorous, serum cholesterol, as well as liver and kidney functions were assessed. A decrease in weight loss was observed in the iontophoretic group compared to respective subcutaneous group with identical conditions. The possible reasons may be stress and side effects due to anesthesia twice a week for the iontophoretic groups. However, this would not be seriously concerned since anesthesia is not necessary for humans who independently adopt iontophoresis. Meanwhile, people can adjust iontophoretic parameters like current density as their demands and the administration time is easily turned on and off. Despite statistically significant decreases in iontophoretic-liposomal PRP treated groups at both high and low doses, the changes in serum calcium were relatively small (0.4~0.5 mg/dL within the normal range of 5.3–13.0 mg/dL) and should not cause pathological effects. These results are coherent with the finding of decreased serum calcium in the patients received PRP four times per day in the previous study although the mechanisms are not clear [42]. Enhancement in intestinal calcium absorption and correction of calcium balance was also shown [42] when using PRP in humans previously to convince our proposal that slight decrease in serum calcium should not lead to pathological effects. However, larger decreases in serum phosphorus were found in low-dose liposomal-PRP/pure PRP iontophoretically and subcutaneously treated groups. The reasons might be related to the demand of phosphorus for increased body bone formation caused by anabolic effects of PRP on bones. Supplement of calcium and vitamin D is necessary as routine osteoporosis treatment to prevent hypocalcemia and hypophosphatemia when the effective low-dose liposomal-PRP is iontophoretically administered. In addition, a mild increase in BUN in I-0.5PRP/LP group coupled with slight decreases of creatinine in iontophoretic-liposomal PRP/pure PRP administered groups might be due to inhibitory actions of PRP on β-adrenergic (β1 and β2-adrenergic) receptors on renal arteries resulting in a slight decrease of blood volume renal arteries and increase in reabsorption of blood urea nitrogen.

## 4. Methods

### 4.1. Materials

Cholesterol, octadecylamine, 1,2-distearoyl, l-α-phosphatidylcholine (DSPC, molecular weight (MW): 790.15 Da), propranolol hydrochloride (MW: 295.8 Da), chloroform, and methanol were obtained from Sigma (St. Louis, MO, USA). All chemicals used in this study were of reagent grade.

### 4.2. Production of DSPC Liposomes

The liposomes were prepared by evaporation sonication method with some modification [43]. Briefly, the phospholipids used for the liposomes were a mixture of DSPC, cholesterol and octadecylamine at the molar ratio of 1:1:0.5. Powder form of DSPC, cholesterol and octadecylamine was dissolved in methanol: chloroform (1:1, *v*/*v*) and loaded into round bottom flask. The flask was placed in laminar flow hood and air dry for 24 h to form a thin layer of film. Nitrogen gas was further used to remove any remaining trace of organic solvent. Rehydration of the thin film with deionized water or PRP solution and followed by 20 minutes of sonication.

### 4.3. Experimental Animals and Pre-Clinical Study Design

**Figure 6:**
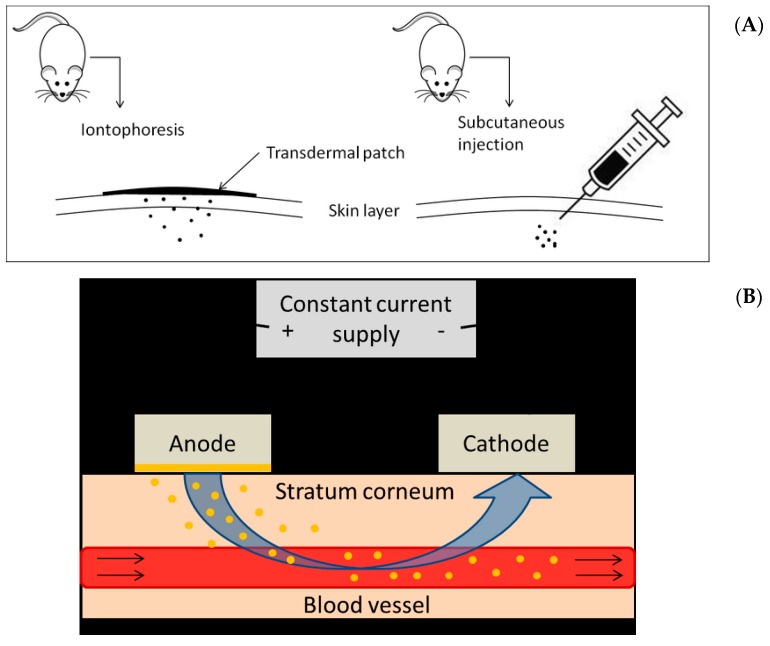
(**A**) Schematic diagram comparing iontophoresis and subcutaneous injection of PRP; (**B**) The theoretical principle of iontophoresis in this study.

The animal use protocol has been reviewed and approved by the Institutional Animal Care and Use Comitee (IACUC), approval number IACUC 103111. A total of 97 female Spray-Dawley (SD) rats used in this study were purchased from BioLASCO (Taipei, Taiwan). The animal study was approved by the Institutional Animal Care and Use Committee (IACUC, approval number: 103111). Rats were kept in a temperature controlled room (±25 °C) and raised on a 12-h light/12-h dark cycle (lights on at 6:00 AM). Food and water were provided ad libitum throughout the experiment. At three months of age, bilateral ovariectomy was performed under anesthesia using Zoletil^®^ (50 mg/kg, i.p.) and xylazine (10 mg/kg, i.p.). Three months after OVX (age of rats: 6 months), the animals were divided into different experimental groups, categorized by PRP concentration, pure compound or with liposomal carriers and delivery methods (as shown in Table 5). Two types of drug delivery pathway, subcutaneous injection and iontophoresis, were adopted. In vivo anti-osteoporotic experiments were performed for 3 months. A schematic diagram was plotted to show the differences between the two administrative methods (Figure 6A) and the general principle of iontophoresis (Figure 6B). At the end of experiment (animal aged 9 months), the weight of rats was measured and blood was collected for serum biochemical analysis. Following on, rats were euthanized by an overdose of CO_2_. In all rats, tibias and spines were excised and cleared of fat and connective tissue, followed by immediately fixed in 10% formaldehyde.

### 4.4. Iontophoretic Parameters

Pure PRP and liposomal PRP at high (0.5 mg/kg) and low (0.05 mg/kg) doses were administered via transdermal iontophoretic delivery or subcutaneous injection. The voltage and current for iontophoresis were generated by a current power generator (GS610, Yokogawa, Japan) combining a set of transdermal patch (PF 383 and PF 384, Perimed, Järfälla, Sweden). The current density of 0.1 mA was applied to stimulate the permeation of PRP or PRP encapsulated liposomes. The location of anode and cathode was placed according to the investigation on β-blocker iontophoresis accomplished by Tashiro et al. [44]. The duration of each experiment was conducted for 2 h per day, 2 days per week, for 12 weeks. The doses of PRP were based on the previous description [37,38] and the well-known potentials of liposomes to decrease administrative doses. The treatment duration and current density used in this study were determined on the basis of our previous findings of PRP release from its liposomes-encapsulated form and the iontophoretic liposomal PRP protocol by Conjeevaram et al. [27,45].

### 4.5. Serum Biochemical Analysis

Serum samples were harvested and sent to Union Clinical Laboratories (UCL, Taipei, Taiwan) for biochemistry tests including analyses of SGOT, SGPT, BUN, creatinine, and electrolytes calcium, inorganic phosphorous as well as cholesterol.

### 4.6. Measurement of Bone Porosity by Micro-Computed Tomography (Micro-CT)

**Figure 7:**
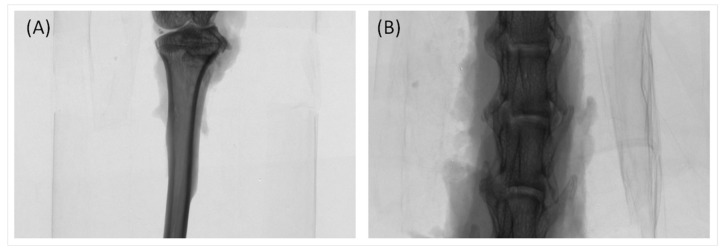
Micro-CT scanning areas of proximal tibia (**A**) and the fourth lumbar section of spine (**B**) in the OVX rats.

The proximal tibia and the fourth lumbar spine of rats (Figure 7) were scanned with a high-resolution micro-CT (Skyscan 1076, Bruker, Belgium). The operating condition was set at a source voltage of 50 kV and a current of 200 mA. Data were acquired at every 0.5° rotation step through 180°. The micro-CT scanning width was set as 34 mm as the height was 17 mm. After scanning, images were re-constructed using CT-analyser (CTAn) computer software (Skyscan, Bruker, Belgium) to assess the trabecular microstructure starting from the region of interest in the binary images. With defined and established threshold in the gray-scale, CTAn software analyzed bone morphometric data such as the ratio between bone volume (BV) and total tissue volume (TV) (BV/TV; %). Trabecular thickness (TbTh; μm), trabecular number (TbN; mm^−1^), trabecular separation (TbSp; μm) and BMD were also determined. The coefficients of variation used for morphometric parameters of BV/TV, TbN, TbTh and TbSp were 2.0%, 1.1%, 0.66%, and 1.30%, respectively, based on the methods in our previous studies [31,46].

### 4.7. Statistical Analysis

Bone morphometric data, BMD and serum biochemical values obtained from each experimental group are presented as mean ± standard error of mean. Results were statistically analyzed using two-way analysis of variance (ANOVA) with Tukey–Kramer multiple comparisons test on SPSS version 17.0 (SPSS Inc., Chicago, IL, USA), to determine whether there were significant differences between the respective control category and experimental group (* *p* < 0.05, ** *p* < 0.01 and *** *p* < 0.001).

## 5. Conclusions

The current study provides convincing evidence that low-dose (0.05 mg/kg) PRP encapsulated within DSPC liposomes in collaborated with iontophoretic administration twice a week caused stable and greatest enhancement in bone formation in proximal tibia and lumbar spine. The present study uncovered that the significance of cooperation of liposomal encapsulation and iontophoresis accomplishes effects and efficiency of PRP on bone formation at lower dose and with longer administrative duration than its current clinical application, previous therapeutic data in rodents and those exerted in combination of pure or liposomal PRP with subcutaneously injective routes. PRP given through liposomal forms and iontophoretic route is able to enhance bone formation at lower applied dosage and with longer administrative duration to pharmacologically reduce its side effects and elevate its potential for clinical protection against bone loss. In order to stably, safely and efficiently display anti-osteoporotic actions in vivo, the data demonstrated that the optimal manner for PRP is liposomal encapsulation collaborated with transdermal iontophoresis.

## Figures and Tables

**Table 1:** Bone measurements in trabecular bone of proximal tibia in the ovariectomized (OVX) rats administered with pure or liposomal propranolol (PRP) by iontophoresis (*n* = 13) or subcutaneous injection (*n* = 8).

Tibia	I-OVX	I-0.05PRP	I-0.5PRP	I-0.05PRP/L	I-0.5PRP/L	S-OVX	S-0.5PRP	S-0.05PRP/L	S-0.5PRP/L
BV/TV, %	4.07 (0.45)	3.41 (0.69)	3.27 (0.41)	9.00 ^a^ (2.04)	2.61 (0.39)	3.78 (0.80)	5.65 (0.63)	7.60 (2.01)	4.77 (0.79)
TbTh, mm	0.10 (0.00)	0.10 (0.00)	0.10 (0.01)	0.11 (0.00)	0.10 (0.00)	0.11 (0.01)	0.11 (0.00)	0.12 (0.01)	0.11 (0.01)
TbN, mm^−1^	0.41 (0.05)	0.29 (0.05)	0.32 (0.04)	0.81 ^a^ (0.19)	0.27 (0.04)	0.41 (0.09)	0.53 (0.07)	0.68 (0.19)	0.45 (0.08)
TbSp, mm	1.10 (0.11)	1.16 (0.07)	1.22 (0.13)	1.04 (0.14)	1.26 (0.08)	1.26 (0.22)	1.09 (0.12)	1.09 (0.22)	1.35 (0.16)

All value presented as mean ± standard error (SE), ^a^ *p* < 0.05, compared to respective OVX group; All groups undergo ovariectomy. I-OVX: control of iontophoresis groups; S-OVX: control of subcutaneous injection groups. TbTh: trabecular thickness; TbN: trabecular number; TbSp: trabecular separation.

**Table 2:** Bone measurements in the trabecular bone of lumbar spine in the OVX rats administered with pure or liposomal PRP by iontophoresis (*n* = 13) or subcutaneous injection (*n* = 8).

Spine	I-OVX	I-0.05PRP	I-0.5PRP	I-0.05PRP/L	I-0.5PRP/L	S-OVX	S-0.5PRP	S-0.05PRP/L	S-0.5PRP/L
BV/TV, %	30.51 (1.90)	26.13 (1.29)	27.60 (1.18)	35.93 ^a^ (1.35)	32.00 (1.31)	31.93 (2.79)	34.73 (1.95)	30.61 (2.51)	31.57 (2.37)
TbTh, mm	0.16 (0.01)	0.14 (0.00)	0.14 (0.00)	0.18 ^b^ (0.01)	0.17 (0.00)	0.18 (0.01)	0.17 (0.01)	0.16 (0.01)	0.16 (0.01)
TbN, mm^−1^	1.92 (0.11)	1.84 (0.08)	1.93 (0.07)	2.01 (0.07)	1.94 (0.08)	1.81 (0.16)	2.01 (0.10)	1.85 (0.08)	1.93 (0.09)
TbSp, mm	0.33 (0.03)	0.34 (0.02)	0.31 (0.01)	0.29 (0.01)	0.32 (0.02)	0.32 (0.03)	0.31 (0.01)	0.31 (0.01)	0.31 (0.02)

All value presented as mean ± SE, ^a^ *p* < 0.05, ^b^ *p* < 0.01, compared to respective OVX group. All groups undergo ovariectomy.

**Table 3:** Effects of pure PRP and liposomes-encapsulated PRP at high and low doses administered with iontophoresis (*n* = 13) or subcutaneous injection (*n* = 8) on serum calcium, inorganic phosphorous and cholesterol level in the OVX rats.

Groups	Calcium (mg/dL)	Phosphorous (mg/dL)	Cholesterol (mg/dL)
Iontophoresis			
I-OVX	11.34 ± 0.13	10.60 ± 0.54	122.92 ± 6.29
I-0.05PRP	11.01 ± 0.11	8.00 ± 0.29 ^c^	115.23 ± 4.54
I-0.5PRP	10.62 ± 0.40	11.72 ± 0.57	123.85 ± 5.98
I-0.05PRP/L	10.69 ± 0.13 ^b^	7.79 ± 0.55 ^b^	124.62 ± 6.25
I-0.5PRP/L	10.88 ± 0.10 ^b^	11.34 ± 0.57	125.31 ± 4.03
Subcutaneous			
S-OVX	11.11 ± 0.13	13.95 ± 1.28	130.38 ± 7.34
S-0.5PRP	11.61 ± 0.23	13.10 ± 1.34	124.88 ± 4.54
S-0.05PRP/L	10.66 ± 0.08 ^a^	7.36 ± 0.36 ^c^	113.13 ± 5.74
S-0.5PRP/L	11.21 ± 0.13	15.76 ± 0.77	137.25 ± 8.17

All value presented as mean ± SE, ^a^ *p* < 0.05; ^b^ *p* < 0.01; ^c^ *p* < 0.001, compared to respective OVX group. All groups undergo ovariectomy. Reference value for calcium is 5.3–13.0 (mg/dL), for phosphorous is 11.0–14.3 (mg/dL), and for cholesterol 40–130 (mg/dL).

**Table 4:** Effects of pure PRP and liposomes-encapsulated PRP at high and low doses administered with iontophoresis (*n* = 13) or subcutaneous injection (*n* = 8) on serum glutamic oxaloacetic transaminase (SGOT), serum glutamic pyruvate transaminase (SGPT), creatinine and blood urea nitrogen (BUN) in the OVX rats.

Groups	SGOT (U/L)	SGPT (U/L)	Creatinine (mg/dL)	BUN (mg/dL)
Iontophoresis				
I-OVX	144.92 ± 13.80	66.85 ± 5.93	0.52 ± 0.01	17.81 ± 0.61
I-0.05PRP	134.38 ± 11.11	54.38 ± 2.64	0.45 ± 0.02 ^b^	17.97 ± 0.51
I-0.5PRP	163.08 ± 12.85	61.15 ± 4.47	0.47 ± 0.01 ^a^	19.34 ± 0.71
I-0.05PRP/L	106.85 ± 9.80 ^a^	58.00 ± 4.22	0.46 ± 0.01 ^b^	17.76 ± 0.54
I-0.5PRP/L	184.69 ± 10.52 ^a^	58.31 ± 3.05	0.45 ± 0.02 ^b^	19.65 ± 0.41 ^a^
Subcutaneous				
S-OVX	185.75 ± 19.16	71.00 ± 9.67	0.44 ± 0.01	16.80 ± 1.04
S-0.5PRP	137.00 ± 17.61	73.13 ± 4.75	0.48 ± 0.01	18.53 ± 0.52
S-0.05PRP/L	191.38 ± 17.11	69.50 ± 4.59	0.45 ± 0.01	16.84 ± 2.24
S-0.5PRP/L	182.38 ± 15.15	70.50 ± 3.65	0.44 ± 0.02	15.18 ± 1.39

All value presented as mean ± SE, ^a^ *p* < 0.05; ^b^ *p* < 0.01, compared to respective OVX group. All groups undergo ovariectomy. Reference value for SGOT is 10–301(U/L); for SGPT is 46–72 (U/L); for creatinine is 0.2–0.8 (mg/dL), and for BUN is 15–21 (mg/dL).

**Table 5:** Experimental groups and their abbreviations.

Treatment	Iontophoresis (*n* = 13)	Subcutaneous (*n* = 8)
OVX	I-OVX	S-OVX
OVX + PRP (0.05 mg/kg)	I-0.05PRP	NA
OVX + PRP (0.5 mg/kg)	I-0.5PRP	S-0.5PRP
OVX + PRP (0.05 mg/kg)/liposome	I-0.05PRP/L	S-0.05PRP/L
OVX + PRP (0.5 mg/kg)/liposome	I-0.5PRP/L	S-0.5PRP/L

*n*: number of rats in respective experimental group.

## Supplementary Materials

Supplementary materials can be found at www.mdpi.com/1422-0067/18/4/822/s1.
