# Supplementary material for: Liposomal Encapsulation for Systemic Delivery of Propranolol via Transdermal Iontophoresis Improves Bone Microarchitecture in Ovariectomized Rats
Source: Int J Mol Sci. 2017 Apr 13;18(4):822. doi: 10.3390/ijms18040822 (PMC5412406; doi:10.3390/ijms18040822)
Supplement: Supplementary file 1 [file ijms-18-00822-s001.pdf]

Supplementary Materials: Liposomal Encapsulation for Systemic Delivery of Propranolol via Transdermal Iontophoresis Improves Bone Microarchitecture in Ovariectomized Rats

Benjamin Teong, Shyh Ming Kuo, Wei-Hsin Tsai, Mei-Ling Ho, Chung-Hwan Chen, Han Hsiang Huang

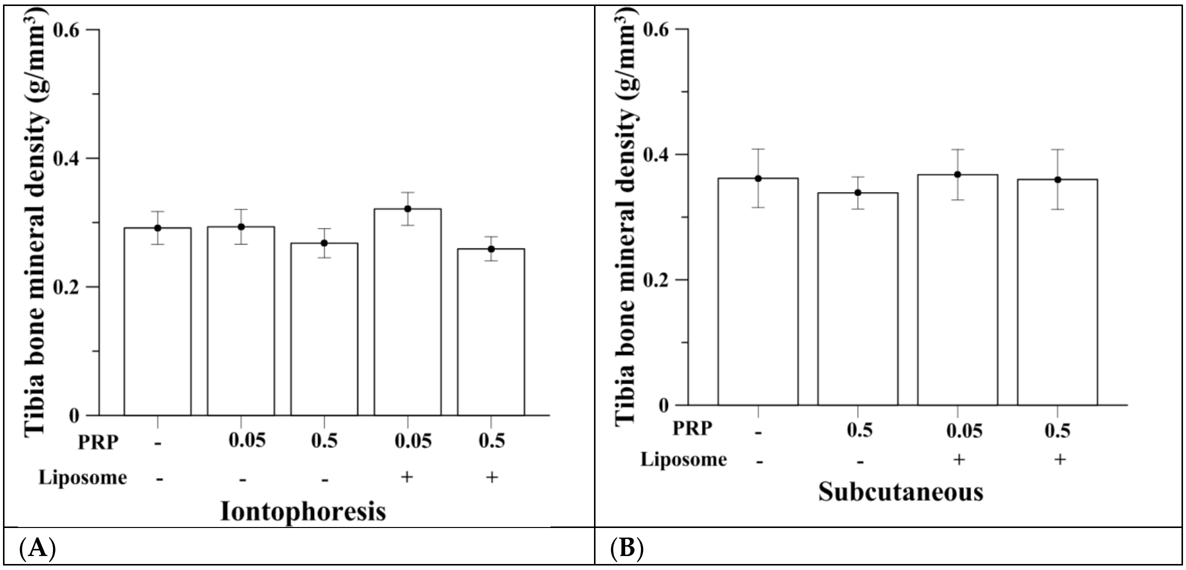

Figure S1. (**A**) Low-dose liposomal propranolol (PRP) (0.05 mg/kg) administered by iontophoresis (n = 13) slightly and insignificantly increased bone mineral density (BMD) in tibia; (**B**) Subcutaneous injection (n = 8) of pure or liposomal PRP did not show significant effects on tibia BMD.

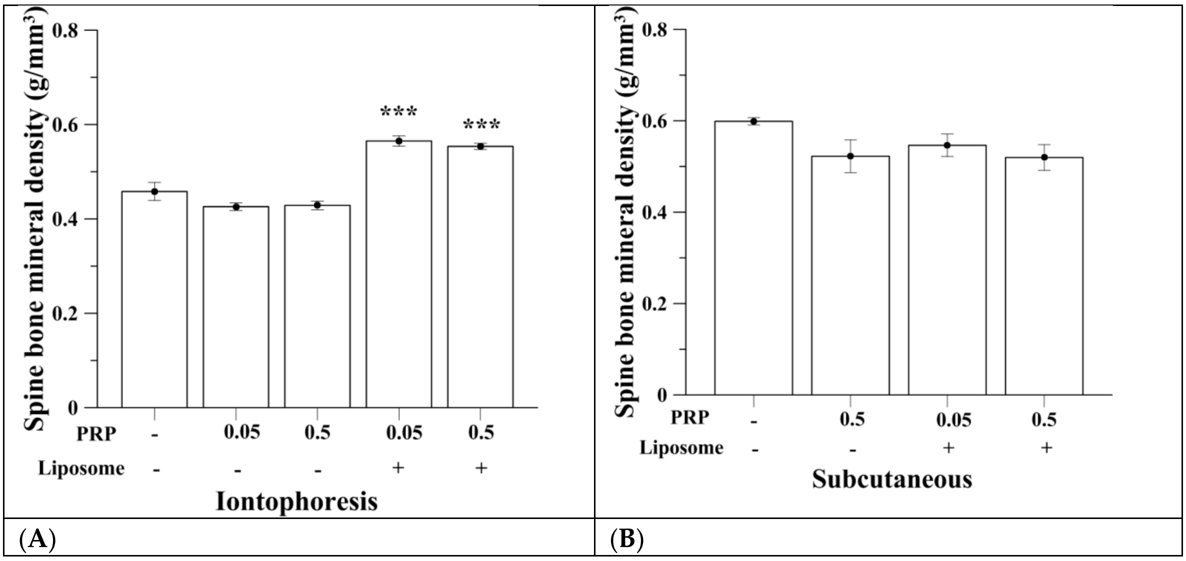

Figure S2. (**A**) Low-dose (0.05 mg/kg) and high-dose (0.5 mg/kg) liposomal PRP given by iontophoresis (n = 13) enhanced BMD in spine (p < 0.001); (**B**) Subcutaneous injection PRP of pure or liposomal forms (n = 8) did not have significant effects on spinal BMD.
